# Charge reversal nano-systems for tumor therapy

**DOI:** 10.1186/s12951-021-01221-8

**Published:** 2022-01-10

**Authors:** Peng Zhang, Daoyuan Chen, Lin Li, Kaoxiang Sun

**Affiliations:** 1 School of Pharmacy, Key Laboratory of Molecular Pharmacology and Drug Evaluation (Yantai University), Ministry of Education, Collaborative Innovation Center of Advanced Drug Delivery System and Biotech Drugs in Universities of Shandong, Yantai University, 30 Qingquan Road, Yantai, 264005 Shandong People’s Republic of China; 2 State Key Laboratory of Long-Acting and Targeting Drug Delivery System, Shandong Luye Pharmaceutical Co. Ltd, Yantai, 264003 People’s Republic of China

**Keywords:** Charge reversal, Stimuli-responsive, Nano systems, Surface charge, Antitumor therapy

## Abstract

Surface charge of biological and medical nanocarriers has been demonstrated to play an important role in cellular uptake. Owing to the unique physicochemical properties, charge-reversal delivery strategy has rapidly developed as a promising approach for drug delivery application, especially for cancer treatment. Charge-reversal nanocarriers are neutral/negatively charged at physiological conditions while could be triggered to positively charged by specific stimuli (i.e., pH, redox, ROS, enzyme, light or temperature) to achieve the prolonged blood circulation and enhanced tumor cellular uptake, thus to potentiate the antitumor effects of delivered therapeutic agents. In this review, we comprehensively summarized the recent advances of charge-reversal nanocarriers, including: (i) the effect of surface charge on cellular uptake; (ii) charge-conversion mechanisms responding to several specific stimuli; (iii) relation between the chemical structure and charge reversal activity; and (iv) polymeric materials that are commonly applied in the charge-reversal delivery systems.

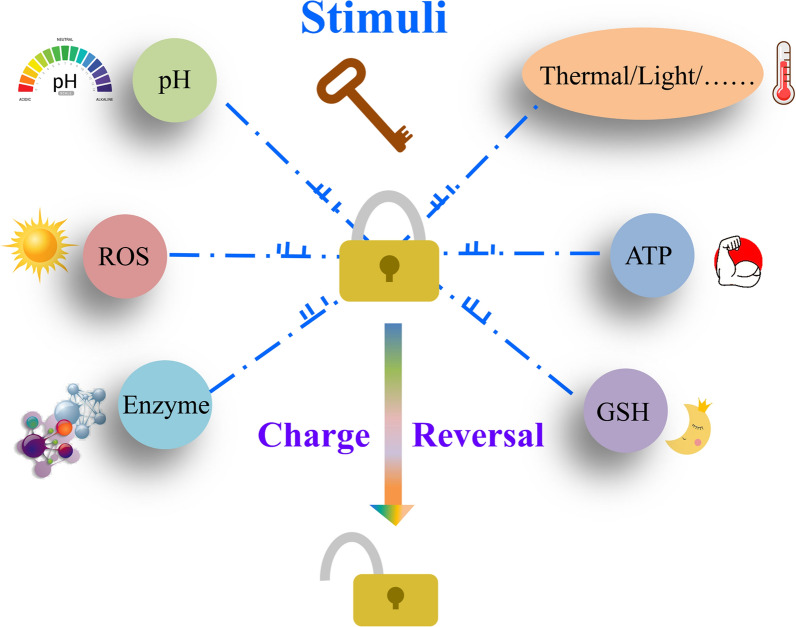

## Introduction

Cancer has become a major public health problem worldwide, according to the data from GLOBOCAN database, about 19.3 million new cancer cases and 10.0 million deaths were reported in 2020 [1]. At present, there are many types of cancer treatment, such as surgery, chemotherapy, radiation therapy and immunotherapy. Chemotherapy is the most often used to treat various types of cancer and chemotherapy drugs have been applied successfully to extend the life of cancer patients. However, the lack of selectivity, poor bioavailability, myelosuppression and multidrug resistance problems are still the major challenges in developing effective chemotherapeutics for efficient clinical cancer treatment [2–5].

Nanotechnology shows potential to overcome the limitations of conventional chemotherapeutics [6]. For the past decades, nano drug delivery systems (NDDSs) are one of the most promising strategies for controlled and targeted drug delivery [7]. Due to the unique physicochemical and biological properties of nanoscale mater, NDDSs exhibit series of clinical advantages, such as targeted delivery, decreased adverse side effects, enhanced anti-inflammatory, improved bioavailability and stability, prolonged plasma exposure and so on [8–10]. For these reasons, numerous types of NDDSs have been designed and shown significant potential in cancer diagnostics and treatment, such as polymeric nanoparticles, liposomes, and micelles [11–13]. However, despite the extensive research and promising results, it is important to note that the fate of the nanocarriers inside the biological system is essential for biomedical application. Recently, with the further understanding about tumor microenvironment (TME) and nano-related physicochemical properties, it’s verified that the inherent properties of NDDSs, for example, the size, shape, surface chemical moieties and surface charge, are highly critical for the cytotoxicity, biodistribution, and internalization of NDDSs to achieve satisfactory therapeutic efficiency [14–16].

Here, we focus on the influence of surface charge of NDDSs on the drug delivery efficacy, and explain how the surface charge plays a vital role for regulating the chemical stability in biological environment and antitumor activity of NDDSs. For the further development of NDDSs benefiting from surface charge, charge reversal NDDSs (CR-NDDSs) were successfully applied and confirmed could enhance the therapeutic efficacy from numbers of studies [17–22]. Thus, different stimuli-responsive strategies for fabricating CR-NDDSs are discussed, including intra- or extracellular signals of TME, such as pH, redox potential (glutathione (GSH)), reactive oxygen species (ROS) and enzymes, as well as materials used in related applications. The purpose of this review is to offer a thorough understanding of the recent progress of the charge-reversal strategy for enhanced antitumor efficacy, that possibly provide potential solutions to facilitate the clinical translation of relevant cancer nanomedicine in the future.

## Effect of surface charge on the uptake of nanocarriers

For the application of NDDSs, since therapeutics must be taken up by tumor cells to achieve therapeutic efficacy of cancer treatment, cellular uptake profile reflects the delivery efficiency and the bioavailability of nanocarriers [23–25]. Among various physicochemical properties of NDDSs, one of dominant factors is the surface charge which control various biological responses to delivery systems. Studies have demonstrated that the electrostatic interactions between the charged nanocarriers with the cytomembrane are of great importance for the cellular uptake, positively charged nanocarriers usually achieved better internalization and higher cellular uptake efficiency than negatively charged ones [26–28]; however, the positive surface charge did trigger the rapid clearance of NDDSs from blood circulation at the same time. The electrostatic attraction between the negatively charged albumin and those positively charged nanoparticles is the main reason for the clearance of cationic nanocarriers [29, 30]. It is generally accepted that the normal blood pH range is 7.35—7.45, and the isoelectric point of most proteins in blood is less than 7, which means that most proteins in the blood are negatively charged [31, 32]. For example, the isoelectric point of human serum albumin (HSA) is 4.7 and it is negatively charged in blood circulation. Therefore, the positively charged nanocarriers would strongly associate to the negative proteins via electrostatic attraction, which would also affect how nanoparticles interact with cells (or uptake mechanism) and influence biodistribution of NDDSs [33–35]. While, neutral nanoparticles or slightly negatively charged nanoparticles could resist the protein adsorption for a longer time [36]. It should be noted that once nanoparticles contact with biological environments, they are modified by adsorption of biomolecules on their surface, which is called protein corona. Formation of protein corona modifies various physicochemical properties of nanoparticles, such as like surface charge, size, aggregation state etc. and these properties would directly or indirectly affect biological activities like pharmacokinetics, therapeutic efficacy, and endocytosis mechanisms for cellular uptake of the nano-nano systems. Although it was suggested that positively charged nanoparticles attract primarily negatively charged proteins, the research evidence indicate that biological environments admit mostly negatively charged corona covered nanoparticles [37]. On one hand, as cytomembrane is negatively charged due to the surface anionic chemical entities, the positively charged nanocarriers could benefit from the high adhesion with cell membranes, which result in enhanced cellular uptake because of the strong electrostatic interaction between charged nanoparticles and cell membranes. On the other hand, the cationic nanocarriers may also promote the clathrin-mediated endocytosis route and/or caveolae-mediated endocytosis route, which are dominant pathways for cellular internalization of NDDSs [38]. However, the role of surface charge of positive particles on cellular uptake is still controversial. For example, positively charged magnetic nanoparticles were better internalized in human breast cancer cells than negative ones, but they did not show obvious internalization influence by human umbilical vein endothelial cells [39]. Protein corona is one possible explanation for this result. Furthermore, positive surface charge can induce serious side effects, the mentioned strong interaction with serum components in the blood and non-specific adhesion with normal cells may result in hemolytic side effects and tissue/cell toxicity [40, 41].

## Mechanisms of triggered surface charge conversion

**Scheme 1:**
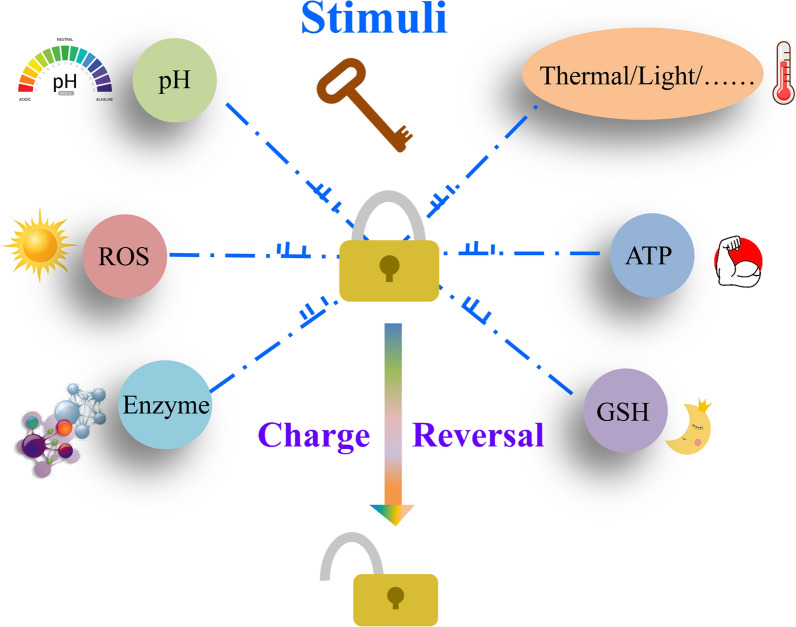
Illustration of the stimuli-responsive CR-NDDSs

The charge conversion process of CR-NDDSs mostly depends on the change of chemical structures of nanocarriers, such as protonation/deprotonation, bonds cleavage, molecular structural variation and so on, which are triggered by internal or external specific stimuli. Based on the reported stimuli, CR-NDDSs can be classified into pH, ROS, enzyme, GSH, ATP, light, and thermal-responsive charge reversal systems. Moreover, many dual or multi stimuli-responsive CR-NDDSs have also been studied so far (Scheme 1).

### pH-Responsive CR-NDDSs

Among all the biological stimuli, pH is the most frequently applied stimulus to trigger charge conversion, because of the pH difference between the physiological blood environment and TME. In general, tumor environment is more acidic (pH 6.5–6.8) than normal tissues (pH 7.15–7.45), while the pH condition of endo/lysosomes is even lower (pH 4.5–5.0) [42]. There are two main typical reported approaches to achieve the pH-triggered charge conversion: i) cleavage of acid-labile bonds; ii) the protonation/deprotonation of surface groups in nanocarriers.

#### Cleavage of acid-labile bonds

The breakage of chemical bonds would lead to the changes of physicochemical properties of NDDSs. Some covalent bonds are typically sensitive to the acidity of environment, including hydrazine, imine, amide, ether, ketal, oxime bonds and so on, one typical example is *β*-carboxylic amide bond [43, 44].

**Fig. 1:**
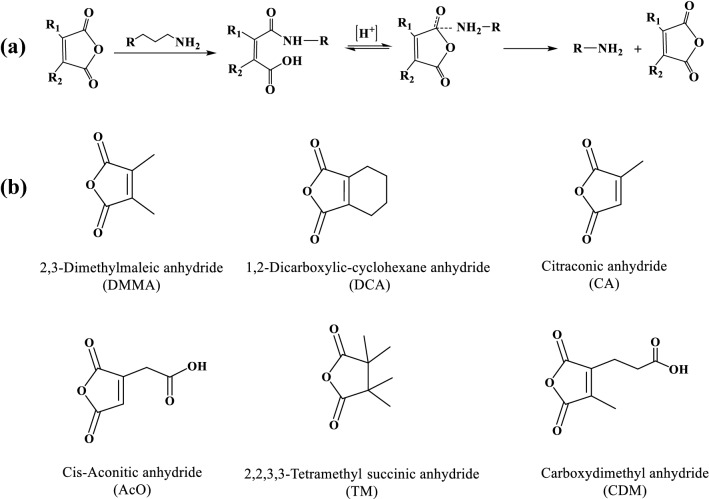
**a** Synthetic scheme and pH-responsive structure change of maleic anhydride derivatives. **b** Some reported chemical structures of *β*-carboxylic anhydrides [48, 49]

The amide bonds are generally stable under most conditions, however those amide bonds containing carboxyl groups on their *β*-positions (in short *β*-carboxylic amide bonds) are pH-sensitive, and could be hydrolyzed into corresponding amine derivatives and anhydrides or dicarboxylic acids at acidic condition [44]. The *β*-carboxylic amide bonds derivatives are usually negatively charged at neutral pH condition due to the presence of the carboxyl groups, but with the degradation of the amide bonds at acidic pH and recovery of primary amino groups which can be protonated at acidic condition, the host compounds undergo a negative-to-positive charge conversion process [45]. It was found that the hydrolysis rate of *β*-carboxylic amide bonds mainly depends on the substituents on the double bonds of anhydride structure, especially the rigidity of grafted anhydride [46]. Normally, substituents and double bonds on the *α, β*-position could facilitate the hydrolysis profile of *β*-carboxylic amide bonds (Fig. 1a) [47].

Shen et al. [48] compared the hydrolysis profiles of 2,3-dimethylmaleic anhydride (DMMA), 1,2-dicarboxylic-cyclohexene anhydride (DCA), and tetramethyl succinic anhydride (TM) amidated primary amine groups of polylysine (PLL). As the primary amine groups regenerate from hydrolysis of the amide bonds, the nanocarriers would convert to positively charged gradually. Therefore, the more rapid charge conversion indicates the more pH sensitivity and faster hydrolysis rate of the amides. At pH 7.4, zeta-potentials of PLL-DMMA, PLL-DCA, and PLL-TM were about -24 mV, -23 mV, and -45 mV at beginning, then PLL-DMMA became positively charged in 10 h, while PLL-DCA and PLL-TM remained negative zeta potential even after 24 h. By contrast, the charge-reversion time was shortened obviously at pH 6.0, the conversion only occurred in 0.5 h for PLL-DMMA, 7.5 h for PLL-DCA, and 30 h for PLL-TM from negative to positive. Further in pH 5.0 condition, the negative-to-positive conversion finished in only 10 h for PLL-TM, whereas 1.5 h for PLL-DCA and converted immediately for PLL-DMMA. Other studies have revealed the influence of substituents on the pH sensitivity of *β*-carboxylic amides. Lee compared degradation kinetics of five maleic acid amide derivatives (maleic acid amide, citraconic acid amide, *cis*-aconitic acid amide, 2-(2'-carboxyethyl) maleic acid amide and 1-methyl-2-(2'-carboxyethyl) maleic acid amide) under different pH conditions at 37℃ [49]. Their results showed that the degradation of amides highly depended on the substituents on the *cis*-double bond and the acidity of environment. As the intramolecular cyclization of *β*-carboxylic amides—to form a five-membered ring—were enhanced by the substituents, and the increased number of substituents could lead to accelerated degradation rate. Besides, extreme acidic environment (low pH value) would apparently facilitate the cyclization reaction and the degradation rate of the amides. Some reported structures of *β*-carboxylic anhydrides were shown in Fig. 1b [48, 49].

Based on the charge-reversal strategy of using *β*-carboxylic amides as acid-labile linkers, several CR-NDDS have been constructed. A hierarchical tumor acidity-responsive magnetic nanobomb (termed HTAMNs) was fabricated for cancer diagnostic imaging and photodynamic therapy [50]. HTAMNs, composed mPEG-block-poly (dopamine-ethylenediamine-DMMA)-L-glutamate-chlorin e6 (Ce6) and superparamagnetic iron oxide NPs, were negatively charged at the physiological conditions thus prolonged the blood circulation time and promoted accumulation of HTAMNs at tumor site based on the enhanced permeability and retention (EPR) effect. Once arrived the tumor extracellular environment, HTAMNs responded to the tumor extracellular pH (pHe) and the *β*-carboxylic acid amide bonds in polypeptide ligands were quickly hydrolyzed to re-expose amine groups, accompanied by the surface charge of HTAMNs converted from negative to positive, resulting in improved cellular uptake, and further enhanced diagnostic imaging sensitivity and photodynamic therapeutic efficacy in human hepatoblastoma xenograft tumors.

Besides 2,3-dimethylmaleic acid amide bonds, other similar structures are also applied in the charge-convertible drug delivery systems, such as citraconic acid amide bonds [51, 52], 1,2-dicarboxylic-cyclohexene acid amide bonds [53], carboxydimethyl acid amide bonds [54] and so on. Due to the structure of *β*-carboxylic amide linkages, all these structures are sensitive to pH and the negative part could be dissociated in acidic environment, thus the surface zeta potentials convert to the positive. Another typical acid-labile amide group is 1,2-dicarboxylic-cyclohexene acid amide bonds (NH-DCA), because of the hydrolysis of NH-DCA in the acidic tumor microenvironment, positively reversed nanocarriers were able to enhance adhesion to the cell membrane. Chang et al.developed a charge-reversal amphiphilic pillar[5]arene system (P5NH-DCA), to kill the cancer cells via cell membrane disruption [53]. Owing to the DCA moieties, the negatively charged P5NH-DCA was stable and unfavorable binding to the cell membranes. Once in the acidic environment, the charge-reversal product P5NH3 containing primary amino groups—upon DCA hydrolysis dissociated—was able to interact with the cancer cell membrane and disrupt the cell membranes.

Besides *β*-carboxylic amides, imine bonds can also be applied to fabricate CR-NDDSs, for example benzoic imine bonds [55, 56]. Benzoic imine bonds, which are formed by primary amines and benzaldehyde groups, remain stable at physiological pH environment but can undergo cleavage under acidic condition. Yang et al. designed a pH multistage responsive block copolymer, poly(ethylene glycol)-benzoic imine-poly(γ-benzyl-L-aspartate)-b-poly(1-vinylimidazole) (PPBV), to deliver paclitaxel and curcumin to breast cancer stem cells (bCSCs) [56]. By introducing the benzoic imine bonds (pH-sensitive linkage), PPBV could de-shield its PEG layer (shield the positive charge), with switching the surface charge from neutral to positive, and reducing its size at tumor site, thus facilitating the cellular uptake and deep tumor penetration.

**Fig. 2:**
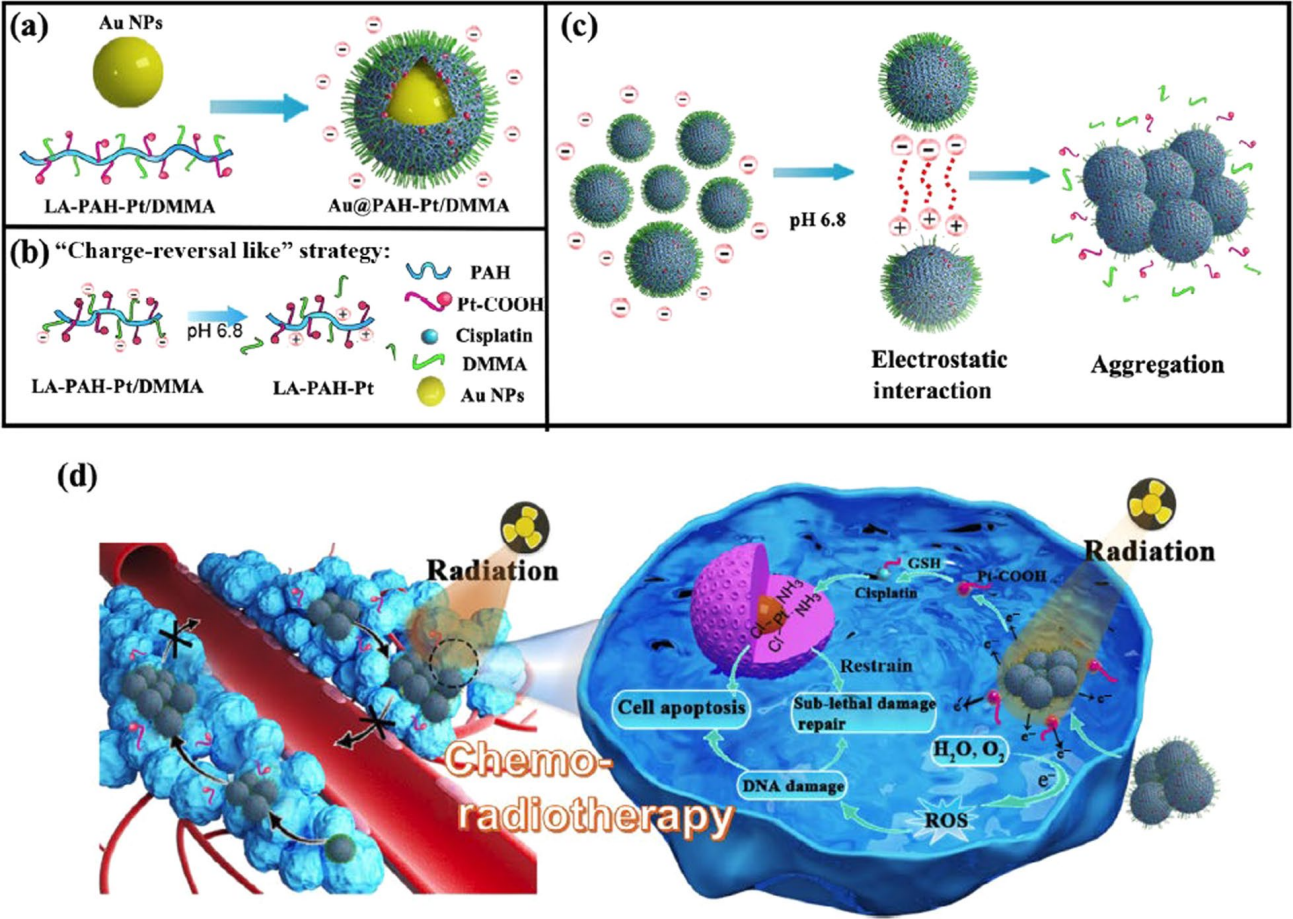
pH-responsive charge-reversal nanoparticles (Au@PAH-Pt/DMMA) to enhance tumor therapeutic efficiency:** a** composites of Au@PAH-Pt/DMMA;** b** mechanism of charge conversion of Au@PAH-Pt/DMMA;** c** electrostatic aggregation;** d** in vivo working behaviors of Au@PAH-Pt/DMMA combined chemo-radiotherapy. Reprinted from Ref. [57] with permission from Springer

In addition, acid-labile bonds may not only act as pH-responsive linkers for CR-NDDSs, but can also serve as hydrophobicity regulator. In one example, a self-aggregating nanosystem Au@poly(allylamine) hydrochloride–cisplatin/DMMA (Au@PAH-Pt/DMMA) was established for combined chemo-radiotherapy [57]. In this system, DMMA was introduced to achieve irreversible aggregation of Au@PAH-Pt nanoparticles. In the acidic TME, the DMMA shell fell off by responding to the pH and exposed the protonated amino groups, the positively charged nanoparticles can promote cellular uptake to enhance chemotherapy effect (Fig. 2). At the same time, with the hydrolysis of DMMA layer, which made Au@PAH-Pt/DMMA hydrophilic in physiological environment, the residual nanoparticles became hydrophobic. Consequently, the self-aggregation process was promoted by both the hydrophobic interaction and electrostatic interaction (between the negatively charged unhydrolyzed particles and positively protonated amidogen via the dissociation of DMMA). This “charge-reversal like” induced aggregation strategy was found to notably increase the cellular uptake of cisplatin and lead to superior antitumor effect.

CR-NDDSs can be fabricated using acid-labile bonds, while they can also be designed via shielding the positive inner by anionic polymers bearing acid-labile bonds, which could be shed off under acidic environment. This part will be discussed in later section.

#### CR-NDDSs with protonation/deprotonation of polymers

**Fig. 3:**
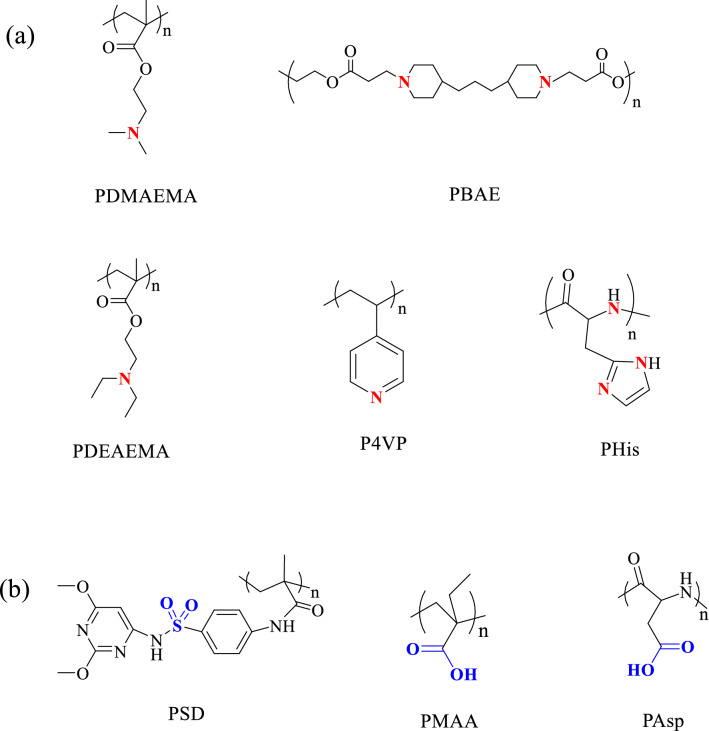
Examples of pH-responsive protonation/deprotonation polymers. **a** cationic polymers and **b** anionic polymers. (According to the Ref. [63–73].* PDMAEMA* poly(2-dimethylaminoethyl methacrylate),* PBAE* poly(beta-amino ester),* PDEAEMA* poly(2‐(diethylamino)ethyl methacrylate),* P4VP* poly(4-vinylpyridine), * PHis* poly(histidine),* PSD* poly(methacryloyl sulfadimethoxine),* PMAA* poly(methacrylic acid),* PAsp* poly(aspartic acid)

Protonation refers to the process of the addition of protons to atoms or molecules, thereby forming their conjugate acids; while deprotonation is the opposite process that protons are removed to produce conjugate bases. Both the protonation and deprotonation of polymers could induce the change in surface zeta potential, whereas the protonation/deprotonation of some groups (such as amino, imidazole sulfonamide, carboxyl groups, etc.) occurs dynamically in physiological pH conditions [58]. When the pKa value of surface protonatable groups below physiological pH condition, NDDSs exhibit negatively charged with deprotonated state; on the contrary, positively charged with protonated state is exhibited [59]. Compared with acid-labile bonds containing systems, CR-NDDSs with protonation/deprotonation of polymers display faster response to pH changes since no breakage of chemical bonds is involved in protonation process. Some examples of pH-responsive protonation/deprotonation polymers are summarized in Fig. 3.

##### CR-NDDSs with pH-responsive Cationic polymers

Cationic polymers are polymers bearing the positive charge or incorporating cations in the structure. Some cationic polymers, especially the cationic centers of which are imidazole groups or amino esters, can be deprotonated at basic/neutral pH, whereas become protonated and hydrophilic under acidic condition [60–62]. For example, owing to the ampholytic nature, carboxymethyl chitosan (CMCS) displayed negative charge at near-neutral media due to the deprotonation of amine and carboxyl groups, the negatively charged carboxymethyl chitosan could reduce plasma protein binding thus to prolong blood circulation time and improve tumor accumulation of nanoparticles. Furthermore, the acidic microenvironment of tumor would induce the protonation of amino groups of CMCS, and the positively converted charge could improve cellular uptake of nanocarriers via electrostatic interaction with the anionic cell membrane [60]. In addition, some protonatable functional groups of polymers not only serve as cationic centers, such as primary and/or secondary amine and imidazole groups, these groups could also serve as model for the endo-/lysosomal escape moieties. A multi-sensitive prodrug micelle [63], consisting of poly(2-(diethylamino)ethyl methacrylate) (PDEA) as protonatable branch for the charge switch, was developed as vehicle for cellular and mitochondria-targeted co-delivery of camptothecin (CPT) and porphyrin derivative photosensitizer for the cervical cancer therapy. The protonation of tertiary amine groups of PDEA in intracellular acidic environment not only triggered the charge reversal from negative (-12 mV) to positive (14 mV), but also facilitated endo-/lysosomal escape to cytoplasm through “proton sponge mechanism”.

Among cationic polymeric vectors for drug and/or gene delivery, poly(beta-amino esters)s (PBAEs) is one of promising candidates because of its high efficiency of DNA and/or siRNA transfection, and low toxicity compared with other cationic polymers such as polyethylenimine (PEI) [64]. In addition, the protonation of tertiary amine groups in PBAEs at weakly acidic condition would lead to the conversion of surface charge, making PBAEs as suitable candidate in CR-NDDSs [65, 66]. Several PBAEs-based nano platforms have been formulated for antitumor drugs delivery, for example lipid-PBAE-HA@DOX [66] with PBAE as charge reversal layer and HA as cluster of differentiation-44 (CD44) binding shell for targeted delivery of DOX to A549 cells, and the in vivo antitumor experiment demonstrated that lipid-PBAE-HA@DOX NPs significantly improved survival in tumor-bearing mice. Poly(histidine) (PHis) is a polypeptide that contains imidazole groups, which the pKa value is 6.5. Li et al. reported PHis-based CR-NDDSs for co-delivery vascular endothelial growth factor siRNA (VEGF siRNA) and etoposide to treat metastatic non-small cell lung cancer [67]. Under blood circulation, histidine moieties provide negatively charged shell, which shield the positive charge to improve the blood circulation stability of system. After accumulation in tumor tissues via EPR effect, the acidic microenvironment triggered the protonation of imidazole groups in histidine, resulting in the charge reversal from negative to positive, which improved penetration into the deep tumor and enhanced cell internalization.

##### CR-NDDSs with pH-responsive anionic polymers

Compared to cationic polymers, anionic polymers could minimize or avoid the nonspecific interactions with serum albumin or proteins thus to improve the stability of NDDSs in the blood circulation [68]. Some anionic polymers, bearing sulfonyl or carboxyl groups, can be deprotonated at neutral pH; but upon reaching acidic condition, the sulfonyl or carboxyl groups would accept protons, thus the surface charge of NDDSs are converted into positive to improve tumor uptake [58].

There are two primary strategies for achieving charge reversal when applied anionic polymers in CR-NDDSs: (i) the direct protonation of anionic polymers under TME [69]; (ii) the positively charged agent-loaded core was exposed via electrostatic repulsion against protonated anionic polymer shield (core/shell cross-linked CR-NDDSs) [70, 71]. It should be noted that the latter is more flexible in constructing CR-NDDSs since the shell and core can be designed and obtained separately.

Nanoparticles with size-shrinkable property can achieve the deeper penetration by changing into a smaller size in the tumor. Jia et al. reported programmed nanoparticles with the variable size strategy and charge-reversible property at the same time for improving tumor treatment efficacy [72]. A pH-sensitive anionic polymer was synthesized (poly(2-ethyl-2-oxazoline)-poly(methacryloyl sulfadimethoxine, PEPSD, pKa = 6.96) for shielding the positive charge of polyamidoamine/doxorubicin (PAMAM/DOX) core. At the physiological environment, PEPSD and PAMAM/DOX can form nanoparticles via electrostatic adsorption. While in the tumor environment, the PEPSD was rapidly protonated to convert the charge from negative to positive, leading the detachment of PEPSD from the nanoparticles to expose positively charged PAMAM/DOX ultrafine nanoparticles. Results confirmed that the tumor accumulation and internal penetration was effectively improved by this inversion of size and charge strategy. Shielding/deshielding strategy can be used in different NDDSs, such as liposomes. Chen et al. prepared a charge-conversional liposomal system to enhance cancer therapeutic efficacy [73]. The cationic liposomes containing Cypate, DOX and NH_4_HCO_3_ were shielded by pH-sensitive poly(methacryloyl sulfadimethoxine) (PSD) through electrostatic interaction at pH 7.4. At the tumor site, PSD was deshielded and the liposomes displayed pH-sensitive charge reversal capability. In vivo results implied that this pH-sensitive charge reversal liposomes could efficiently enhance the tumor accumulation, antitumor efficacy, and reduce systemic side effects of DOX.

##### CR-NDDSs with pH-responsive zwitterionic polymers

Zwitterionic polymers, containing of both anionic and cationic terminal groups, exhibit high resistance to non-specific protein adsorption [74]. Due to this advantage, zwitterionic polymers could escape the recognition and clearance of the immune system in the body. At the same time, zwitterionic polymers can be endowed with pH-responsive charge convertible feature by controlling the ratio of anionic and cationic groups to change their isoelectric point [75–77].

**Fig. 4:**
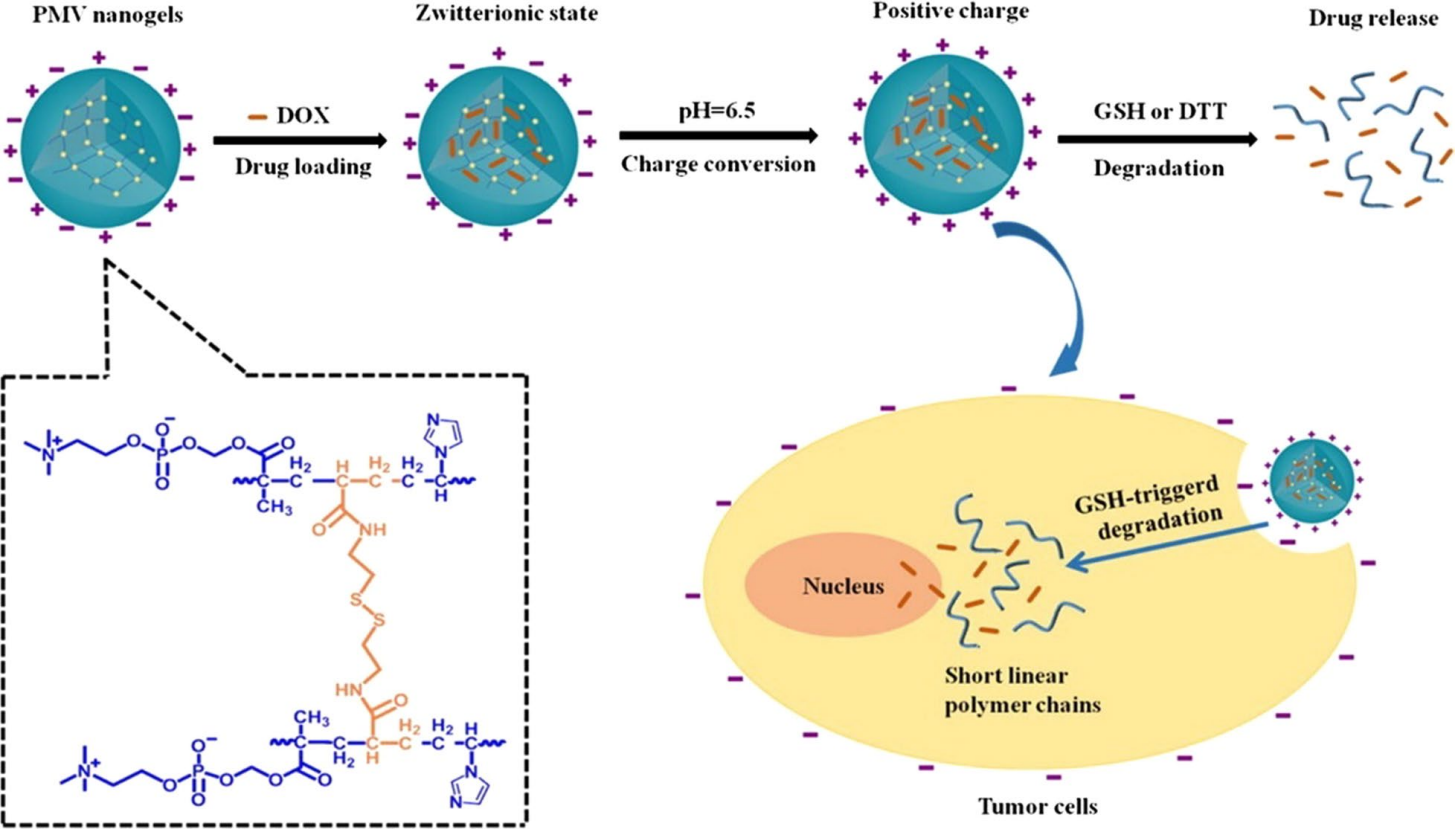
Illustration of the charge-conversion ability and GSH-triggered biodegradable behavior of PMV zwitterionic nanogels. Reprinted from Ref [78]. with permission from Elsevier

A pH-sensitive nanogel, [poly(2-methacryloyloxyethyl phosphorylcholine-s–s-vinylimidazole)] (p(MPC-ss-VIM), PMV) was fabricated to delivery DOX for non-small cell lung cancer therapy (shown in Fig. 4) [78]. This nanogel exhibited rapid positive charge switch behavior at tumor extracellular pH environment due to the protonation of the imidazole ring of poly(vinylimidazole), which rapidly protonated at pH 6.5. Owing to the anti-fouling property of zwitterionic state, PMV showed great protein-adsorption resistance at pH 7.4, and the circulation time of PMV nanogel was significantly prolonged. Furthermore, PMV nanogel achieved promoted tumor cell internalization because of its charge-conversion ability. On the other hand, by precisely controlling the ratio of anionic and cationic components, zwitterionic nanoparticles can rapidly change their surface charge at the isoelectric point. For instance, a boronate ester crosslinked zwitterionic nanogel (NGCA) was designed by Zhang and coworkers, by adjusting the ratio of the amino and carboxyl moieties in the nanogels, negative-to-positive surface charge conversion of NGCA can be achieved at tumor extracellular pH, which enhance their cellular uptake efficiency [79].

### Enzyme-responsive CR-NDDSs

As a key component of the bio-tools, enzymes play a central role in series of bioactivities and cell regulations. Some enzymes are found overexpressed in various tumor tissues, such as hyaluronidase [80], *β*-glucuronidase [81], *γ*-glutamyl transpeptidase [82], esterase [83] and so on. By incorporating specific enzymatic substrates into the NDDSs, those carriers can be recognized and cleaved by the target enzymes. Thus, enzyme-triggered CR-NDDSs are able to be engineered to change the structure on demand by responding to the intracellular and/or extracellular overexpressed enzymes, accompanying with the surface zeta potential switch [84]. Considering that enzymatic activation usually occurs in specific region of tumor tissue, enzyme triggered CR-NDDSs are highly promising strategies for targeted drug delivery, which show great potential in long-term cycling and reduced adverse effects to healthy cells and tissues.

*β*-glucuronidase (*β*-G), a member of glycosidase family, catalyses the hydrolysis of *β*-D-glucuronic acid residue. *β*-G is generally located in the endosomes/lysosomes and overexpressed in the tumor extracellular microenvironment. By grafting *β*-glucuronic acid to the primary amine of staramine, Sun et al. [19] engineered a charge-reversal lipid pro-staramine (namely GluAcNA) to achieve prolonged circulation and mitochondria-targeted delivery of anticancer drugs. When exposed to *β*-G in tumor extracellular environments, the capped *β*-glucuronic acid in GluAcNA can be enzymatically hydrolyzed and the nanocarriers converted to the positively charged staramine, thereby facilitating endocytosis of GluAcNA.

**Fig. 5:**
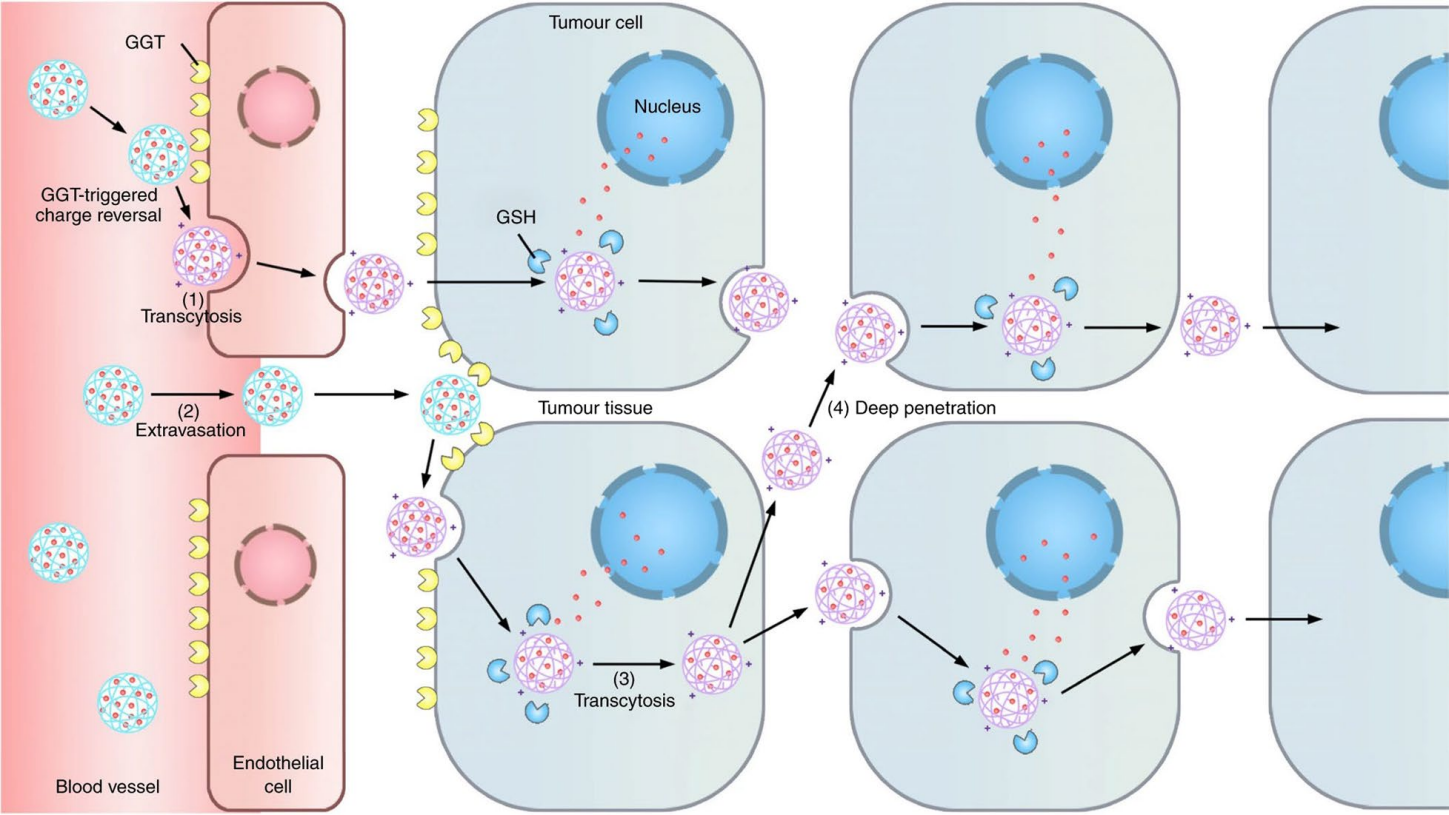
Illustration of the behavior of GGT-responsive nanomedicine: (1) stable in neural, (2) nanoparticles extravasate into the tumor interstitium, (3) active tumor penetration, (4) rapid internalization via the adsorption-mediated transcytosis. Reprinted from Ref. [85] with permission from Nature

Not only *β*-glucuronidase, *γ*-glutamyl transpeptidase (GGT) can also act as stimuli to trigger the charge reversal process [84]. It has been found that GGT is overexpressed in several human tumors, and GGT cleaves the *γ*-glutamyl amide bonds to generate primary amines. Shen et al. [85] has developed a GGT-responsive conjugate to deliver camptothecin into pancreatic tumor cells (shown in Fig. 5). Once the conjugate entered tumor blood vessels or tumor interstitium, the overexpressed GGT hydrolyzed the *γ*-glutamyl moieties to regenerate positive primary amines. Subsequently, the positively charged conjugate underwent caveolae-mediated endocytosis and transcytosis, achieving transendothelial and transcellular transport of the cationic conjugate to enhance tumor penetration and anticancer efficacy.

As a biocompatible, biodegradable and nonimmunogenic material, hyaluronic acid (HA) serves as a major ligand of CD44 (cluster of differentiation 44) and has been widely utilized in therapeutics delivery. He et al. reported a combined dual-drug chemo-immune therapy to maximize therapeutic efficacy [86]. In this nanoplatform, HA (the surface layer) could facilitate the targeting of particles to CD44 receptor and be degraded by hyaluronidase (HAase), which are both overexpressed in the tumors. After HA degradation, the inner positively charged drug-loaded particles with smaller size and negative-to-positive charge reversal were released to penetrate deep into the tumor and efficiently internationalized by tumor cells.

### ROS-responsive CR-NDDSs

Due to the high metabolic activity of cancer cells, elevated levels of reactive oxygen species (ROS), including hydroxyl radicals (OH·), superoxides (O_2_·^−^), nitric oxide (NO·) and hydrogen peroxides (H_2_O_2_), have been detected in the intracellular tumor environment compared with normal tissues or tumor extracellular environment [87]. Such differences in ROS level can be employed to trigger charge-reversal process to achieve enhanced antitumor efficiency in cancer therapy [88]. Various ROS-sensitive functional groups have been used for efficient drug delivery, and some of those bonds could also be utilized to construct CR-NDDSs for cancer therapy (shown in Table 1) [20, 89]. In this type of CR-NDDSs, ROS-sensitive chemical bonds are broken-down within ROS-rich intracellular environment and nanocarriers are degraded to achieve charge conversion. For example, boronic acid, one of ROS-mediated oxidizable structures, can be oxidized into tertiary amines by H_2_O_2_. Based on this, Gao and Shen developed a ROS-responsive charge-switchable polymer containing boronic acid benzyl structure for gene transfection of neural stem cells (NSCs) [89]. This ROS-responsive charge-reversal cationic polymer, poly[(2-acryloyl)ethyl(*p*-boronic acid benzyl)diethylammonium bromide] (B-PDEA), could effectively condense brain-derived neurotrophic factor genes into polyplex nanoparticles for effective DNA protection and cellular uptake; after internalization, the intracellular ROS oxidized B-PDEA into negatively charged polyacrylic acid, releasing DNA for expression. Liu et al. reported a ROS-responsive polymer for Alzheimer’s disease therapy [90]. In this study, poly[(2-acryloyl)ethyl(p-boronic acid benzyl) dimethylammoniumbromide] (PDMAEA-BAP, PB) was used for siSTAT3 condensation. Since the elevated ROS level in dysfunctional microglia, PB-siSTAT3 nanoparticles were broken up and cargos were released simultaneously into cytoplasm. Another study from the same research group was glioblastoma treatment by using ROS-responsive nanoparticle for RNAi-based immunochemotherapy [91]. After exposure to ROS environment, the reactive oxygen species can trigger the charge reversal (from positive to negative) by oxidizing benzylboronic acid from BA-PDEAEA and elicit the release of RNAi via electrostatic repulsion.

**Table 1:** Examples of ROS-sensitive linkers applied in CR-NDDSs

ROS-sensitive linkers for CR-NDDSs	Chemical structure and oxidation mechanism	Refs.
Arylboronic ester		[89, 91]
Thioketal		[92]
Peroxalate ester		[93]

Besides boronic ester structure, other ROS-responsive moieties were also reported to inverse the surface charge of NDDSs, for example thioketal and peroxalate ester [92, 93]. Liu and co-workers reported a ROS-sensitive surface charge changeable siRNA vector to overcome the dendrimers toxicity, which was based on the interaction of cationic surface charge of polyamidoamine (PAMAM) dendrimers with negatively charged biological membranes (resulted in membrane disruption and erosion). The thioketal was introduced as linkage to get ROS-sensitive dendrimer (ROS-PAMAM). The low toxicity mechanism of ROS-PAMAM was attributed to its easily cleavable capability in ROS abundant conditions, which reduced the size and surface charge quantity of PAMAM [92]. A programmable vesicular nanodevices based on the triblock copolymer containing poly(ethylene glycol) (PEG) and poly(caprolactone) (PCL) with peroxalate esters (PO) as ROS-responsive linkage (PEG-PO-PCL-PO-PEG), are developed by Li et al. for co-delivery of hypoxia-activated prodrug and glucose oxidase (GOD) [93]. Due to peroxalate esters between PEG and PCL were cleaved by reaction with tumor high level of H_2_O_2_, the negative PEG segments were dissociated from the copolymer, this ROS-sensitive nanovesicle can be activated by the high level of H_2_O_2_ in tumor microenvironment to improve the permeability of membranes.

Although ROS-triggered strategy does have shown potential for tumor therapy, the ROS level of tumor intracellular environment is not sufficiently to induce the cleavage of chemical bonds in very short time. To overcome this obstacle, combinational formulation of ROS-trigger with other strategies, such as pH/ROS dual-sensitive CR-NDDSs, are able to obviously enhance the therapeutic effect. These types of CR-NDDSs are presented in the below section.

### ATP-responsive CR-NDDSs

As the “energy currency” of cells, adenosine triphosphate (ATP) plays fundamental roles in cell metabolism. Meanwhile, ATP also shows great potential for nucleic acid or protein delivery as one of the stimuli-responsive triggers for cancer therapy [94, 95]. Intracellular ATP concentration in the TME (1–10 mM) is more than 1,000-fold higher than in normal tissues, which difference is much higher than that of pH, thus ATP can be used to distinguish the environment between solid tumors and normal tissues [96]. The intra/extracellular gradient of ATP inspired researchers to design ATP-responsive CR-NDDSs. The most developed ATP-responsive CR-NDDSs were mainly based on its unique property to trigger the cleavage of the borate ester bonds, such as phenylboronic acid functional complex [97–101]. Phenylboronic acids (PBA) are able to bind with molecules containing *cis*-diol moieties with high affinity through reversible five-membered ring formation. After a molecule binds with PBA through diol structure, the binded-molecule still can be replaced by another molecule with higher PBA affinity, which tends to form a more stable phenylborate ester structure. The ribose structure with *cis*-diol of the APT has led to a high affinity for PBA, thus inducing triggered molecules (drugs or genes) release due to the exchange reaction with ATP [102–104].

An ATP-sensitive charge reversal nano-platform based on PBA grafted polycation polymer was report for the targeted delivery of anticancer agents [100]. For example, alginate was used as diol linker to construct crosslinked cationic polymers (CrossPPA)/siRNA through crosslinking PBA-tethered PEI (1.8k) with alginate by borate ester bonds. CrossPPA/siRNA was internalized by cancer cells via sialic acid-receptor (a receptor overexpressed on tumor cells which is affinitive to PBA) mediation. After cellular internalization, the borate ester bond between PBA and alginate was broken under intracellular ATP condition, resulting in the disassembly of crosslinked polymer, cationic-to-anionic charge reversal and rapid siRNA release (owing to the electrostatic repulsion between anionic complex and negative siRNA). By using similar strategies, the same research group have constructed ATP-triggered CR-NDDSs with borate ester bonds fraction, such as HA-PEI/siRNA nano system and mPEG-b-poly(2-[(2-aminoethyl)amino]ethylaspatamide) (pDET) nano-platform [98, 105].

Sun et al. developed an ATP-sensitive permeable nanocluster for MRI diagnosis guided photothermal therapy of breast cancer [101]. A charge switchable polycationic carrier, Dextrin-ethylenediamine-phenylboronic acid (Dextrin-EDA-PBA, DEP), was fabricated to modulate the assembly and disassembly of nanoclusters with/without ATP stimulation. In this system, Gd^3+^ and CuS-coloaded small bovine serum albumin nanoparticles (GdCuB) were synthesized and encapsulated into DEP to form DEP/GdCuB nanoclusters. In blood circulation, DEP/GdCuB significantly extended the half-lifetime and thereby enhanced the tumor accumulation of GdCuB. When the clusters reach the tumor site, the extracellular ATP can effectively trigger the release of GdCuB, resulting in tumoral deep penetration as well as the activation of MRI.

### GSH-responsive charge reversal delivery system

Glutathione (GSH), a tripeptide that comprised of glutamine acid, glycine, and cysteine, serves as an important antioxidant in cells. The level of GSH between intracellular cancer cells (2 ~ 10 mM) and extracellular tumor environment (2 –20 μM) is significantly different [106]. By taking advantage of GSH difference between intracellular and extracellular of cancer cells, CR-NDDSs with GSH-sensitive linkages can be designed to maintain the stability of the vehicles during blood circulation, but easily be degraded and release the incorporated drug in the cytosol of cancer cells, resulting in enhanced therapeutic efficacy while reducing the side effects [107–112]. Therefore, GSH-sensitive nano systems are ideal candidates for the development of CR-NDDSs to achieve both targeted drug delivery and enhanced cellular uptake of nanocarriers (Table 2).

**Table 2:** GSH responsive linkers and mechanisms for CR-NDDSs

GSH responsive linkers	GSH-responsive mechanism	Refs.
Disulfide bond		[107, 113]
Diselenide bond		[114]
Ditelluride bond		[112]

Disulfide bonds (*S–S*), which are stable in physiological environments with low level of GSH but rapidly cleaved in tumor intracellular environment through the thiol–disulfide exchange with redox GSH to facilitate the degradation of carriers and the release of cargoes, are commonly used in the GSH-responsive charge-reversal drug delivery systems. For example, Zhang et al.constructed GSH turn-on charge reversal core/shell nanocomplex to achieve increased stability, improved cellular uptake, facilitated endo-lysosomal escape and enhanced antitumor efficacy [113]. This nanocomplex was composed of anionic hyaluronic acid (HA)-disulfide bonds grafted-sensitive outer shell and the cationic PAMAM@DOX core with encapsulated doxorubicin (DOX) into the hydrophobic cavities of polyamidoamine (PAMAM) dendrimers. The anionic outer layer could promote cellular uptake by HA receptor-mediated endocytosis. After internalization into tumor cells, the outer shell of the internalized nanocomplex was disassembled in endo-lysosomes via the destruction of disulfide linkages to re-expose PAMAM@DOX core. This action induced release of the encapsulated DOX and facilitated endo-lysosomal escape through the synergistic action of the proton sponge effect and cationic–anionic interaction between protonated PAMAM and endo-lysosome membranes. In vitro release profiles demonstrated the intracellular environment-responsive release behavior of DOX from this nanocomplex, with a cumulative release of 80% within 4 days in a simulated tumor intracellular microenvironment, whereas the surface charge changed from − 18.82 mV to + 10.95 mV. Cui [107] designed self-assembly of disulfide-containing chitosan oligosaccharide and carboxymethyl chitosan (COS-SS-CMC) with mesoporous silica nanoparticles (MSNs) to deliver DOX (DOX@MSNs-COS-SS-CMC) for cervical cancer treatment. Under mimicking intracellular tumor environment (pH 5.5, 10 mM GSH), DOX@MSNs-COS-SS-CMC were disassembled into particles with a wide size distribution and the surface charges were constantly decreased, suggesting the reduction cleavage of disulfide bonds. Furthermore, in vitro drug release rate of DOX@MSNs-COS-SS-CMC in tumor environments was sevenfold higher than that under normal physiological conditions after 200 h, and the intracellular uptake of DOX@MSNs-COS-SS-CMC was 1.9-fold higher than free DOX extracellular tumor microenvironment (pH 6.5, 10 mM GSH).

Despite many studies about applying disulfide bonds in the GSH-responsive charge reversal delivery systems, diselenide bonds are attracting increasing attention in this field as well. At one side, diselenide bonds (*Se-Se*) show similar redox-responsive activity as *S–S* bonds; on the other side, the bond energy of *Se-Se* bonds (172 kJ/mol) is much lower than that of *S–S* bonds (268 kJ/mol) [115]. Based on these advantages, NDDSs with the application of *Se-Se* have been reported in tumor therapy, including charge-reversal nanoplatforms [114, 116, 117]. He and coworkers [114] developed GSH-responsive prodrug (dimeric paclitaxel nanoparticles, PTXD NPs) containing diselenide bonds for targeted triple negative breast cancer (TNBC) treatment. Diselenide bonds in PTXD NPs were selectively cleaved by the reduced potential in tumor intracellular redox microenvironment to release the prototype drug, which prevented pre-drug leakage in physiological condition. Intrinsic properties of PTXD NPs such as size and surface charge played critical roles in the internalization results. This GSH-responsive nanoparticles achieved efficient triple negative breast cancer accumulation, thus exhibiting stronger anti-tumor efficacy both in vitro and in vivo.

In another study, GSH-responsive ditelluride bonds were utilized to synthesize nanoparticles releasing loaded doxorubicin in cancer cells [112]. Compared to the *S–S* bond, the detelluride bond has even lower bond energy. PEGylated folic acid (FA) and redox responsive ditelluride linkage (-*TeTe*-) were introduced to create a nanosystem with DOX loading. The in vivo tumor treatment study showed this GSH-responsive nanosystem did significantly suppress the tumor growth.

### Other stimuli-responsive CR-NDDSs

In addition to the above-mentioned typical types of stimuli-responsive CR-NDDSs, other strategies and stimuli have also been reported, such as hypoxia-triggered [118], H_2_S-responsive [119], light-triggered and thermo-responsive [120] CR-NDDSs, etc*.*

Efficient ferroptosis therapy mediated by Fenton reaction need low pH environment (pH 3 ~ 4) and sufficient H_2_O_2_. Glucose oxidase (GOx) oxidized oxygen and glucose into H_2_O_2_ and gluconic acid; while conversely, the constant O_2_ consumption could create hypoxia microenvironment that partly impede the ascending intracellular H_2_O_2_ level [121]. Fortunately, 4,4'-azonzenecarboxylic acid (Azo) containing azobenzene group could be specifically reduced by bio-reductases under tumor hypoxia conditions. Base on this principle, Sun et al. [118] constructed an Azo modified zeolitic imidazolate framework (ZIF) biomimetic nanoreactor loading ferric-gallic acid NPs to promote ferroptosis therapy. Owing to the hypoxia conditions resulted from the oxygen consumption by GOx, Azo achieved anionic-to-cationic charge conversion and resulted in enhanced tumor accumulation for positive feedback cellular uptake on MCF-7 cells.

**Fig. 6:**
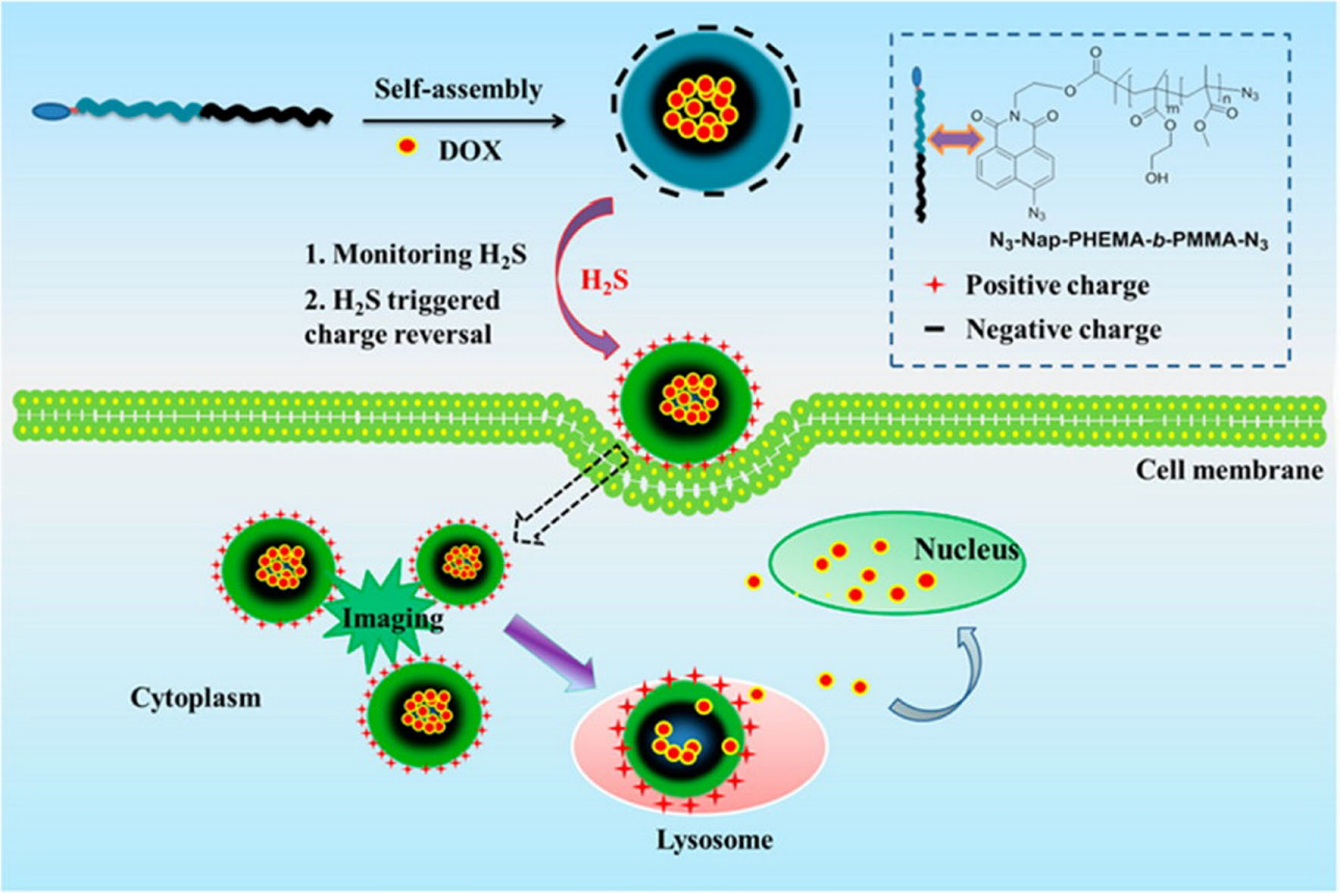
Mechanism of H_2_S-triggered charge reversal and the cell uptake process for co-polymer micelles. Reprinted from Ref. [119] with permission from American Chemical Society

Hydrogen sulfide (H_2_S) is involved in many cancer biological processes. On the basis of the roles of H_2_S in cancer development and progression, Lin et al. [119] fabricated N-(2-hydroxyethyl)-4-azide-1,8-naphthalimide tailed amphiphilic di-copolymer poly (2-hydroxyethyl methacrylate)-block-poly(methylmethacrylate) (N_3_-Nap-PHEMA-b-PMMA-N_3_)@DOX micelles, the negatively charged azido group on the surface of the micelle can be reduced into amide group (positively charged) in the presence of H_2_S, leading to a quick conversion of surface charge. Thus, in tumor site, H_2_S triggered the micelles to occur negative-to-positive charge switch, resulting in facilitated cellular uptake through electrostatic attraction and a fast DOX release (the process was shown in Fig. 6).

### Dual and multi-responsive CR-NDDSs

Compared with single stimulus responsive charge reversal systems, some CR-NDDSs could consecutively respond to different stimuli in extracellular and intracellular environment of cancer cells, such as pH/GSH [122, 123], pH/ROS [88, 124, 125], ROS/Near-Infrared Light [126], pH/Thermal/GSH [127], etc*.* These dual and multi-responsive CR-NDDSs exhibit superior antitumor effects and negligible systemic toxicity, thus have attracted extensive research attention in tumor treatment as well.

By conjugating GSH-responsive 6-mercaptopurine (6MP)-based prodrug and pH-responsive DOX-based prodrug with poly(DEA)-b-Poly(ABMA-co-OEGMA) (PDPAO), Wang et al. constructed pH/GSH-responsive polymeric micelles (M(DOX/6MP)) [122]. It appeared negative-to-positive charge conversion from − 7.29 ± 0.76 mV to 9.31 ± 1.11 mV upon encountering extracellular acidic environment because of the protonation of PDEA, and thus the cellular internalization was facilitated as mentioned before. In intracellular TME (pH 5.0, GSH 10 mM), DOX could diffuse out from hydrophobic core after the cleavage of imine bonds, and rapid 6MP/DOX release was induced. The in vitro evaluation assay of M(DOX/6MP) showed synergistic efficiency cell-killing capability against both HeLa or HL-60 cells. A pH/ROS dual-responsive surface charge-reversal micelles strategy (PCDMA) was reported to deliver podophyllotoxin (PPT) and cucurbitacin B (CuB) to overcome multidrug resistance (MDR) problem, which is one of the main reasons for tumor chemotherapy failure [125]. After arrived tumor tissue, the surface charge of PCDMA could rapidly reverse to positive in the tumor extracellular environment to promote cellular uptake. Subsequently, the PCDMA could be degraded to release PPT and CuB in response to an intracellular high ROS condition. The released CuB is competent for generating ROS, which in turn accelerated the continue release of PPT and CuB. Both the in vitro and in vivo studies demonstrated that PCDMA was effectively internalized by cancer cells and produces massive ROS intracellular, rapid release drug, and effectively killed MDR cancer cells.

**Fig. 7:**
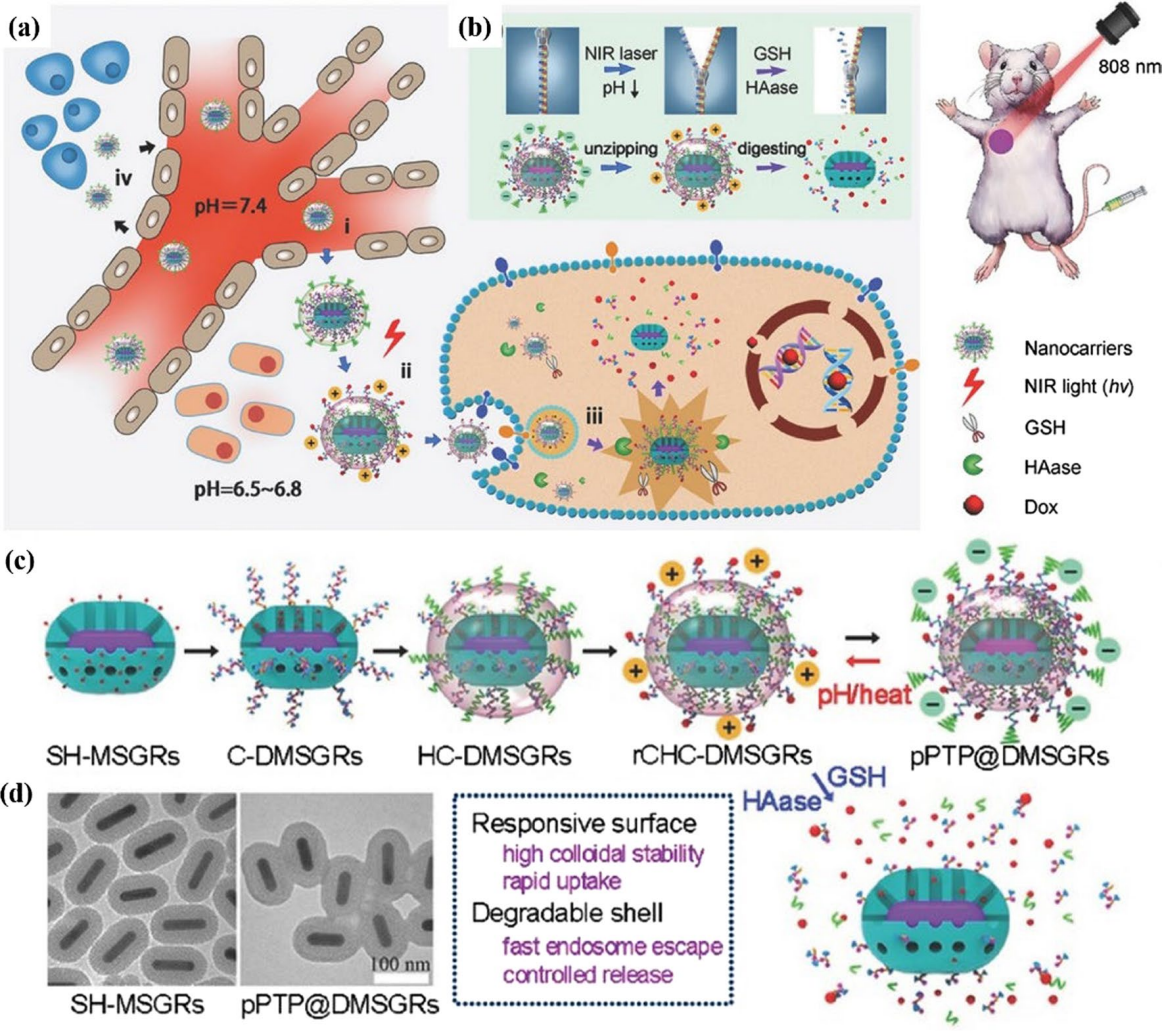
pH-/thermal-/GSH-responsive charge reversal drug delivery system for tumor therapy. **a** charge reversal and drug release process: (i) accumulation via the EPR effect; (ii) NIR-/pH-activated surface change for cellular uptake; (iii) endosome escape and controlled release response to GSH/HAase. **b** Surface charge conversion and polymer shell degradation. **c** Preparation and pH-/thermal-triggered surface charge conversion process. **d** TEM images. Reproduced from Ref. [127] with permission from WILEY‐VCH Verlag GmbH & Co. KGaA, Weinheim

Inspired by the influence of the amino acid residues under acidic microenvironment on the surface charge density of protein, a pH/thermal/GSH multi-responsive system which comprised by cell-penetrating poly(disulfide)s and thermosensitive zipper polymers was designed (shown in Fig. 7) [127]. The polymer was composed of cell-penetrating poly(disulfide)s bearing guanidinium (Gu^+^) residues (CPDs) and thermosensitive polymers bearing phosphate (pY^−^) residues (PTPs), to tailor the surface states for efficient drug delivery. The surface composition and physicochemical properties of this nano system could be tuned at tumor sites, where the acidic microenvironment and photothermal heating broke the pY^−^/Gu^+^ binding to expose the penetrating shell and the surface charge was reversed due to the tuned surface composition, that the decreased pH tended to weaken the binding affinity and the further phase transition via heating caused the zipper rupture. This multi-responsive charge-reversal zipper showed longer blood circulation time, minimized drug leakage, enhanced accumulation and efficient drug release at tumor sites, and significantly inhibited the tumor growth.

## Current development of polymeric materials for CR-NDDSs applications

Charge-reversal drug delivery strategies have experienced remarkable progress over the past years, and polymeric materials contribute in a large extent to such progress. In order to achieve satisfying therapy effects, specific properties of materials are required, including physicochemical stability, biocompatibility, stimuli-responsibility and biodegradability [128, 129]. Now, a number of natural or synthetic polymeric materials have been used to design smart CR-NDDSs, such as poly(ε-caprolactone) (PCL) [130], polysaccharides (particularly hyaluronic acid and chitosan) [131–133], proteins or polypeptides [67], Poly(amidoamine) dendrimer [134, 135], Polyethyleneimine [136, 137], etc*.* In this section, we will outline the current development and performances of natural and synthetic polymeric materials for CR-NDDSs.

### Polysaccharides-based materials for CR-NDDSs

Polysaccharides, or polymeric monosaccharides, are composed of repeating monosaccharides covalently linked through glycosidic bonds. As natural biomaterials, polysaccharides have abundant natural resources, such as from animal (chitosan, chondroitin), plant (*e.g.* cellulose), alga (alginate) and microorganisms (*e.g.* dextran). In addition, most of polysaccharides exhibit excellent biocompatibility, biodegradability, non-immunogenicity, physicochemical stability and versatile functionalization capacity [138]. All these characteristics make polysaccharides ideal biomaterials for the construction of smart delivery systems, that could prolong the residence time and release the entrapped molecules at required time and location, in response to specific physiological stimuli or disease-specific signals [139].

Particularly, polysaccharides have abundant reactive groups on the main chain or side chains, such as hydroxyl, carboxyl or amino groups. Due to these derivable groups, polysaccharides can be chemically modified, resulting in various designed physicochemical properties of polysaccharide derivatives. From the viewpoint of surface charges of special structures or functional groups, polysaccharides can be divided into positively charged polysaccharides (chitosan typically, containing amino groups) and negatively charged polysaccharides (hyaluronic acid, alginate, etc*.*, usually containing hydroxyl and carboxyl groups) [140].

In view of tremendous performance of polysaccharides, various types of polysaccharide-based CR-NDDSs have been applied in biological and biomedical research, especially for cancer therapy. Herein, we present the design and construction strategies of polysaccharide-based CR-NDDSs, and the proceeded research progress in the field of tumor therapy is summarized also.

#### Hyaluronic acid-based materials for CR-NDDSs

Hyaluronic acid (HA) is a linear anionic polysaccharide and contains repeating units of d-glucuronic acid and *N*-acetyl-d-glucosamine through alternating *β*-1,3 and *β*-1,4 glycosidic linkages. Due to the biocompatible, biodegradable, nontoxic, non-inflammatory and non-immunogenic characteristics, HA has been used widely in drug and gene delivery study, especially in cancer treatment research because of its specific targeting capability to CD44 receptor which was overexpressed in several cell types including cancer stem cells [141–143]. Furthermore, the presence of carboxyl and hydroxyl groups in the HA structure makes HA an ideal candidate for chemical modification.

As a type of negative polysaccharide, HA and HA-grafted polymers are generally utilized to shield positive charges and enhance the stability of NPs during the blood circulation process. When responded to the stimulus of intracellular or extracellular environment, HA-grafted layer could be degraded to re-expose the positive constituents, which would facilitate tumor cells internalization and cellular uptake, leading to enhanced drug bioavailability [131, 143–145]. Based on this strategy, a few of CR-NDDSs with different architectures and properties have been developed. Du et al.reported a novel pH-sensitive CR-NDDSs that were able to invert the surface zeta potential under acidic conditions in the presence of HAase [144]. This HA-based charge conversion micelle—constructed by HA, poly(lactic acid) (PLA) and polyamidoamine dendrimers—was applied to delivery docetaxel (DTX) for breast cancer treatment. HA was used to shield the positive charges of PAMAM in order to reduce hemolytic toxicity and cytotoxicity, however, it would be degraded in the presence of HAase and subsequently re-expose the cationic core. Moreover, HA was specifically recognized by CD44 receptors, which could increase intracellular accumulation of DTX. Another charge-switchable micelle, formed via layer-by-layer assembly of cationic protamine (PRM) and anionic hyaluronic acid, was fabricated for delivery of gambogic acid (GA) to overcome drug resistance in human lung adenocarcinoma (A549) tumor cell [145]. In hyaluronidase (HAase)-rich tumor microenvironment, the rapid HA detachment would expose PRM, subsequently lead to surface charge reversal and activation of “proton sponge effect”, which was associated with the endosomal/lysosomal escape. In this work, the zeta potential of HA-PRM-GA micelle maintained negative charge (- 6.28 mV) at pH 7.4, while rose up to + 2.30 mV at pH 6.4 and + 3.52 mV at pH 5.4, accompanied with particle size reduction from 100 to 78 nm after 8 h in PBS buffers containing HAase (2.0 mg/mL). As result of the HA receptor-mediated endocytosis, the enzyme-sensitive structural detachment and proton sponge effect of PRM, HA-PRM-GA micelle showed a remarkable ability to permeate A549 cells, evade endosomes/lysosomes, and ensure the intracellular release of GA.

In an interesting protein/HA study, feather keratin (FK) was used as crosslinker to fabricate FK/HA-based nanogels (DOX@C-FK/HA) as pH-activated surface negative-to-positive charge-reversal protein/polysaccharide complex nanocarriers for the tumor targeting delivery [131]. Due to the breakage of electrostatic interaction between amino groups in positively-charged FK and carboxyl groups in negatively-charged HA with the pH value went down from 8.0 to 4.0, zeta potential of DOX@C-FK/HA raised from − 5.84 mV to + 3.19 mV, which could enhance the cellular internalization of nanocarriers via electrostatic interaction with negative cell membrane. In addition, the thiol groups in the feather keratin could be oxidized into disulfide cross linkage, thus to inhibit the premature drug leakage.

#### Chitosan-based polymeric materials for CR-NDDSs

Chitosan (CS), one of the most abundant polysaccharides and the only natural cationic polysaccharide, is a linear cationic polysaccharide composed of *β*-1,4-linked *N*-acetyl-*D*-glucosamine and *D*-glucosamine units. Chitosan is poorly soluble in water at neutral or basic environment; however, under acidic pH condition, due to the amino groups (-NH_2_) are protonated to form protonated amine groups (-NH_3_^+^), whereby chitosan is converted to soluble polyelectrolyte in acidic media [146].

Since chitosan exhibits excellent biological properties such as nontoxicity, biocompatibility, and biodegradability, it has attracted great attention in the pharmaceutical and biomedical fields. In addition, its polycationic character gives chitosan the ability to bind strongly to several cells that could facilitate cellular uptake and dramatically promote therapeutic efficacy, lead to broad potential applications, including drug and gene delivery [147, 148]. Moreover, Chitosan also has the primary and secondary hydroxyl groups on its backbone, along with the cationic characters, which provide possibility for utilization in surface charge reversal drug delivery technologies.

One of the most preferred chitosan-modification strategies is binding the guest substances to chitosan via covalent bonds [149]. Such chitosan-derived conjugation could maintain the essential properties of chitosan backbones, meanwhile endow chitosan with new properties that ascribed to the grafted bioactive molecules, such as targeting groups or other functional groups/residues which could be desirable to respond to environmental stimuli [132, 150].

For being used as charge reversal drug delivery nanocarrier, the necessary composition of the chitosan-based conjugates should contain therapeutic molecules (small molecule drugs, proteins/peptides, gene, etc*.*), chitosan backbones, and conditionally cleavable covalent bonds. Most of these covalent bonds are designed for cleavage in response to certain endogenous or exogenous stimuli, such as pH conditions [132], redox [133], and so on.

As one of the most commonly used molecules for surface charge reversion, 2,3-dimethylmaleic anhydride (DMMA) could be easily modified to shield the cationic residues of chitosan. At the acidic environment, due to the hydrolysis of the amide bonds between DMMA and CS, the surface charge was rapidly reversed from negative to positive [132, 151, 152]. Jin et al.combined cationic polymyxin B (PMB) with anionic DMMA-CS to get CS-DMMA/PMB for acute lung infection treatment [152]. In bacterially induced acidic pH environment, the negative charged CS-DMMA nanoparticles (− 8.75 at pH 7.4) would turn to positive (+ 2.63 mV at pH 5.5) because of the hydrolysis of *β*-carboxylic acid amide bonds between DMMA and amino group, subsequently released PMB.

**Fig. 8:**
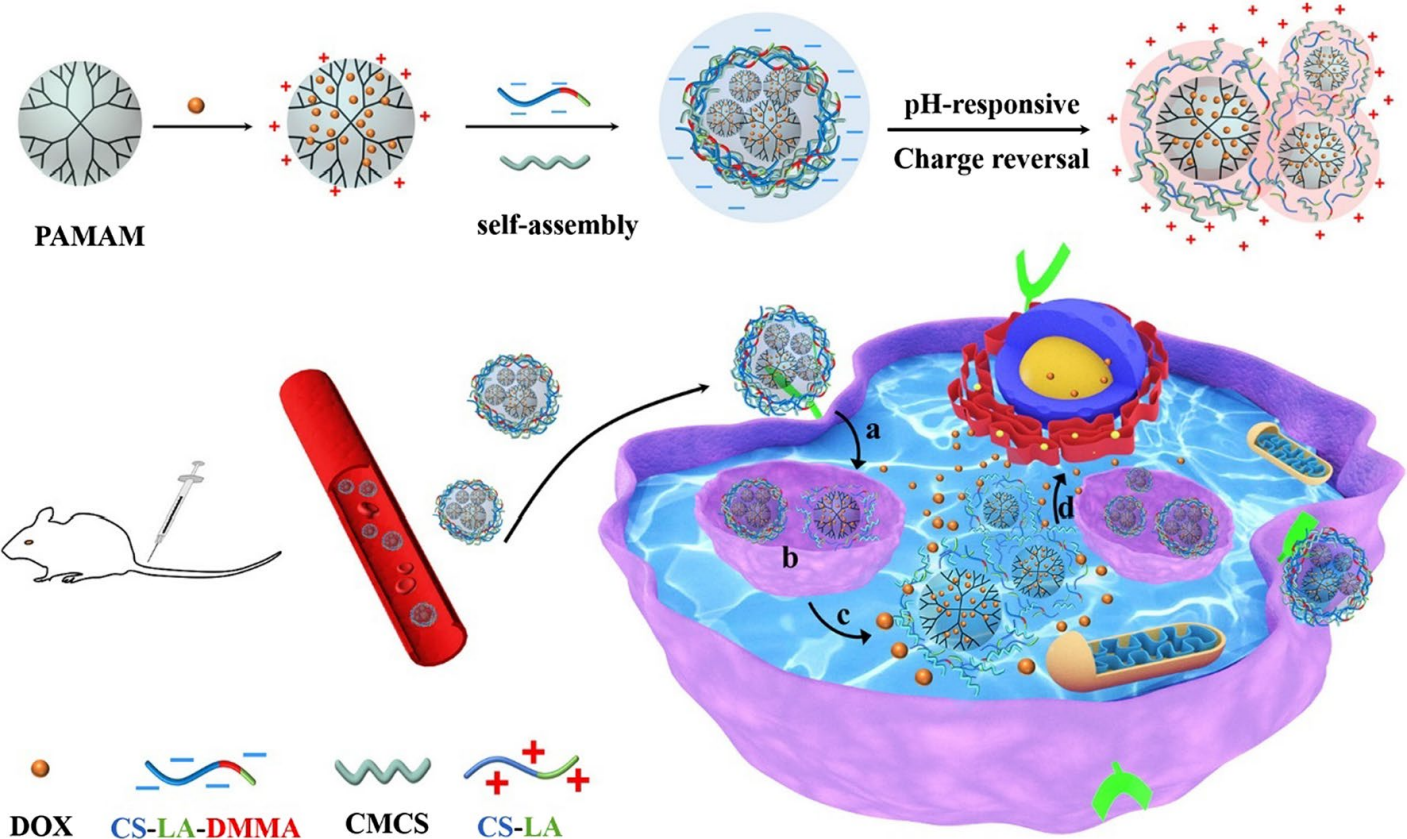
Drug release mechanism and lysosomal escape of chitosan-based nanoformulation within intracellular environment. (**a** cellular uptake via clathrin-mediated endocytosis pathway;** b** lysosomal escape;** c** nanoformulation dissemble and release drug;** d** efficient tumor cell killing). Reprinted from Ref. [132] with permission from Elsevier

Besides the shielding of positive charge by chemical modification, introducing anionic O-carboxymethyl chitosan (CMCS) is the alternative approach to enhance the stability of chitosan-based CR-NDDSs in blood circulation. When pH is less than 6.5, the anionic CMCS (pKa is 6.5 ~ 7.0) would switch to cationic polymer due to the protonation of the amino carboxyl groups. Zhang and co-workers fabricated pH-actived charge reversal core–shell NPs with DOX loaded cationic PAMAM-based nanoparticles (PAMAM@DOX), with CMCS as bridge polymer, DMMA and lactose acid (LA) modified CS as charge-reversal layer (CS-LA-DMMA) for selectively targeting cancer cells [132]. By applying carboxymethyl chitosan as bridge polymer and negatively charged chitosan-derivative as outer shell, the stability and pH-sensitivity of this nanoformulation is promisingly enhanced. Due to the hydrolysis of *β*-carboxylic acid amide bonds under acidic environment, the zeta potential of CS-LA-DMMA switched from − 26.03 mV (pH 7.4) to + 12.56 mV (pH 5.0), and the positively charged PAMAM@DOX was re-exposed via electrostatic repulsion (shown in Fig. 8). Furthermore, the internalized PAMAM@DOX could escape from lysosomes via “proton sponge effect” and “cationic–anionic interaction with lysosome membranes”. Admirable cellular uptake and high apoptosis/necrosis rate were detected in this study. In vitro assays demonstrate that this nanosystem was internalized predominantly via the clathrin-mediated endocytosis pathway [132].

Moreover, there are also polysaccharide-based CR-NDDSs that constructed by combining anionic polysaccharide with cationic polysaccharide, for example, hyaluronic acid and chitosan. In consideration of the different pKa values of HA (pKa is about 3.2) and CS (pKa is about 6.5), HA/CS-based nanoplatform can be designed as negatively charged in physiological environment due to the existence of carboxyl groups of HA. However, under the slightly acidic condition, both the deprotonation of HA and the protonation of CS could change to positive surface charge. Suo et al.[153] applied layer-by-layer self-assembly to synthesize polysaccharide-based nanosystem by consecutively decorating gold nanorods (GNR) with aldehyde/catechol-functionalized HA (DAHA) and hydroxyethyl chitosan (HECS) interlayer as pH-triggered charge-reversal delivery system (GNR-HA^DOX^CH). Carboxyl groups of HA and amino groups of CS reversed the zeta potential from negative to positive in acidic tumor extracellular, then GNR-HA^DOX^CH was internalized into MCF-7 cells via both CD44 receptor and electrostatic interaction dual-mediated endocytosis. Chen and co-workers constructed dual-responsive NPs oHA-PBA@DHPA-CDB/Cur via coating the anionic oligomerichyaluronic acid-carboxyphenylboronic acid (oHA-PBA) out of curcumin (Cur) encapsulated cationic micelles (DHPA-CDB/Cur) [154]. After NPs arrived TME, the pH-sensitive borate ester linkage between PBA and DHPA was broken accompanied with oHA degradation, then underwent negative-to-positive charge reversal to facilitate cellular uptake, mitochondrial targeting and GSH-triggered drug release in PANC-1 cells.

#### Other polysaccharide materials for CR-NDDSs

Besides hyaluronic acid and chitosan, other polysaccharides (*e.g.*, pullulan [43], starch [155], dextran [156], alginate [157]) have also gained much attention in developing CR-NDDSs because of their excellent physicochemical properties.

Pullulan, a linear homopolysaccharide and frequently used coating agent in pharmaceuticals, can be easily derivatized in order to impart new physicochemical properties. Meanwhile, pullulan exhibits affinity to asialoglycoprotein receptor (ASGPR) which overexpressed on hepatoma carcinoma cells [158]. Wang et al. fabricated pH/GSH-sensitive nanoparticles composed of charge-switchable pullulan-based (CAPL) shell and disulfide-containing poly(*β*-amino ester) (PBAE) core for co-delivery of methotrexate (MTX) and gene to HepG2 cells [43]. The nanoparticles exhibited step-by-step responsibility to acidic TME via the cleavage of *β*-carboxylic amide bond in CAPL (for charge conversion) and the “proton sponge” effect of PBAE (to accelerate endo/lysosomal escape). Zeta potential of the nanoparticles changed from -15.1 mV (under pH 7.4) to + 3.5 mV (at pH 6.5) and + 9.5 mV (at pH 5.5) after 2 h, respectively. The cellular uptake by confocal laser scanning microscopy (CLSM) showed that the pullulan-based nanoparticles had enhanced fluorescence signals of DNA after incubation for 4 h and 10 h, which was due to the ASGPR-targeted endocytosis and the electrostatic interaction between nanoparticles and cell membranes.

Starch and starch-derivatives are used as efficient stimuli responsive drug carrier due to their biodegradability, less immunogenicity, and biocompatibility properties. Also, the existences of hydroxyl groups in the side chains of the starch off excellent performance in the pharmacological application [159]. By using aminated starch, negatively charged Helianthus tuberosus—CuO nanoparticles was reversed into positive to improve the cellular internalization of nanoparticles through electrostatic interaction with negatively charged cancerous cell membrane [155].

Zwitterionic nanoparticles with negative surface charges are expected excellent non-specific protein adsorption to prolong blood circulation, enhance permeability and drug delivery efficacy [160]. Ni employed dextran to prepare charge reversal zwitterionic nanoparticles for the DOX delivery [156]. Negative charges were introduced to dextran by the modification of succinic anhydride (SA). Then, the modified dextran (Dex/SA) reacted with cystamine (Cys), which Cys molecules acted as crosslinkers to form zwitterionic nanoparticles. The residual NH_3_^+^ and COO^−^ groups provided particular stability for the zwitterionic nanoparticles in aqueous solution and also the charge-reversal feature. The surface charge was convertible from negative to positive in response to pH changes, thus inducing enhanced cellular uptake due to the strengthened nanoparticle-cellular membrane interaction.

### Polypeptides-based CR-NDDSs

Polypeptides are polymers of amino acids covalently linked through peptide bonds. Similar as polysaccharides, most polypeptides are biocompatible and biodegradable that can be employed as excellent candidates in different applications [160].Notably, some polypeptides with ionizable groups can form electrostatic interactions with oppositely charged molecules, such as DNA, RNA, proteins and charged drugs. Therefore, polypeptide-based materials have gained increasing attention for their intelligent potentials in biomedical and pharmaceutical applications [161]. As materials that contains protonatable and deprotonatable moieties (pKa of α-COOH is 2.3, while pKa of α-NH_3_^+^ is 9.9), polypeptides exhibit changeable net charge that affected by the pH condition. Usually, the polypeptides show net negative charge at pH above the isoelectric pH point (PI) and net positive charge at pH below pI [162]. Based on these characteristics of polypeptide, many promising research studies have been reported in charge reversal strategies for drug or gene delivery [130, 163–165].

Tang et al. [130] employed “delayed charge reversal” strategy to construct polyion complex (PIC) that poly (lysine)-b-polycaprolactone (PLys-b-PCL) micelles as the cationic core with coating the anionic poly (glutamic acid)-g-methoxyl poly (ethylene glycol) (PGlu-g-mPEG) layer. Both the particle size and zeta-potential of this polyion complex showed crosslinking degree-dependent properties. In this study, different charge switch profiles of complex were realized by varying the shell crosslinking degree (CL) of cationic micelle, then controlling electrostatic interaction with PIC layer. Additionally, lower crosslinking degree implied higher chain entanglement and more available -NH_3_^+^ of PLL to electrostatically interact with PIC layer, and displayed lower de-coating and charge-switchable ability. At tumor tissue, PIC with lower crosslinking degree would simultaneously facilitate deeper penetration and higher cellular accumulation.

Besides the protonation and deprotonation strategy, other charge-reversal strategies can also be applied in constructing polypeptide-based CR-NDDSs, for example the acid labile bonds cleavage, such as hydrazone, amide, imine, etc. Huo et al. [165] prepared dual pH-sensitive polymer micelles poly(ethylene glycol)-block -poly(l-lysine) (PEG-b-PLL) for co-delivery disulfiram (DSF) and paclitaxel into multidrug resistant human mammary adenocarcinoma (MCF-7/Adr) cells to overcome multidrug resistance (MDR) challenge. By covalently modified DMMA to poly(L-lysine) (PLL), this nanosystem exhibited negative zeta potential (-13.3 mV) at pH 7.4 environment. However, owing to the hydrolysis of the DMMA group under acidic condition, surface charge of the micelles quickly reversed to + 6.5 mV after 15 min at pH 6.5.

Not only chemical modifications to offer polypeptides with charge-reversal ability, anionic polypeptides can also be directly applied to regulate the surface charge. By adjusting the ratio of modules with opposite charges, Zhang et al.employed “one-pot modular assembly” strategy to develop cytosolic siRNA delivery system which exhibited charge-reversible feature [166]. In this study, Glutamic acid (Glu) was used as anionic module for shielding the positive surface charge of liposome/siRNA complex and endowed the vector with the charge-switchable property, which was due to the protonation of the carboxyl groups in acidic environment.

### Other polymeric materials for CR-NDDSs

When polymers bearing primary amine groups are exposed to low pH environment, the amine end-capping can be protonated, thereby turning them into positively charged polymers [167], such as poly(amidoamine) dendrimer (PAMAM), polyethylenimine (PEI) and so on. These polymers have been extensively explored as drug and gene vehicles because of the abundant surface functional groups for drug conjugation and molecular grafting [168]. Remarkably, the cationic polymers with amino groups ending captured much attention as smart nanocarriers for drug delivery because of the “proton sponge effect”-mediated endosome/lysosome escape properties [169]. However, positive surface charge of those polymers (due to -NH_2_ termini) usually cause a variety of problems for the in vivo applications, including rapid clearance from the blood circulation, hemolysis and systemic toxicity as discussed before [29]. To overcome these obstacles, surface modification strategies for shielding the surface positive charge of cationic polymers-based CR-NDDSs are introduced, and surface charge reversal approach is one of them.

#### Poly(amidoamine) dendrimer

Poly(amidoamine) dendrimer, or PAMAM, with precise numbers of terminal groups and branched structures, are emerging as novel platforms in drug delivery, gene therapy, medical imaging and diagnostic application due to its unique properties, such as multiple functionalities, and size-tunability [168]. However, because of safety problem associated with positive charges on the PAMAM surface, administration of PAMAM dendrimers may result in undesirable effects or toxicity to organs like the liver, spleen, and kidney [170]. Tailoring the surface charge properties provides an opportunity to circumvent the biological obstacles of PAMAM [113, 132].

In the PAMAM-based CR-NDDSs, the shielding/deshielding of the cationic PAMAM core with pH sensitive polymer strategy has been widely applied [134, 135, 144]. By using G4 PAMAM as DOX-conjugating support, He and coworkers established anti-metastatic core–shell nanoplatform for the combination of immunotherapy and chemotherapy [134]. In this delivery system, DOX was coupled to the amino-terminated PAMAM by pH-sensitive hydrazone bonds, and low molecular weight heparin was applied as the coating layer to shield the positive charge of PAMAM-DOX (heparin could also provide anti-metastatic effects). Because of the excessive negative heparin, this nanocarrier was stable and negatively charged but reversed to positively charged when arrived tumor tissue due to the degradation of heparin and exposure of the cationic nanocore, thus the cellular uptake was elevated. Feng et al.also developed a core/shell PAMAM charge conversional nanocarrier with pH/redox sensitive size and charge-changeable properties for anticancer drug podophyllotoxin (PPT) delivery [135]. This nanocarrier was composed of a charge-reversible polymer shell and a redox-sensitive core via electrostatic interactions under normal physiological pH (7.4). The negative shell, which was PEG and dimethylmaleic acid-grafted polyallylamine (PEG-PAH-DMA), was able to maintain negative charge in physiological environment, but revert to positive charge in a mildly acidic tumor environment (pH 6.5), leading to the release of positive PAMAM-drug via electrostatic repulsion. The positive PAMAM-drug core (PPT conjugated with PAMAM through disulfide linkage, namely PPT-ss-PAMAM) was easily to permeate the tumor center and internalized by cancer cells, further released PPT in response to high intracellular concentrations of GSH. Charge reversal of PAMAM-based drug delivery strategy could be employed as non-viral vectors for the gene delivery [143, 171]. By introducing acid-labile acetal groups into PAMAM, a heterogeneous charge-reversal dendrimer was synthesized by Yang and coworkers [171]. The surface moiety of this dendrimer turned from amine-terminated to hydroxyl-terminated due to the cleavage of the acetal groups in the weakly acidic environment, inducing the change of surface charge.

#### Polyethyleneimine

Polyethyleneimine (PEI) is a cationic polymer and has been widely used as non-viral vector for gene transfection. In addition, because of the large number of protonable amino in PEI, it can also serve as the proton sponge to facilitate the endo-/lysosomal escape. However, its clinical translation is severely limited by its safety concern which is related to the positive surface charge effects similarly as PAMAM [172]. To reduce the cytotoxicity as well as further improve the transfection efficiency, different PEI-based systems have been explored.

In one study about the treatment of ulcerative colitis [136], PEI was employed to prepare cationic lipid nanoparticles. The cationic property of PEI lipid nanoparticles (PEI-LNPs) can enhance mucoadhesion to inflamed tissues in the colon and subsequently improve the uptake efficiency by inflammatory cells. However, the stability problem under acidic conditions and premature burst drug release in the stomach are the major limitations for the use of cationic lipid nanoparticles in colon-targeted drug delivery. To overcome the cationic surface challenge in oral colon targeted delivery, Eudragit^®^ S100 (ES) was coated on PEI-LNPs to obtain pH-triggered charge-reversal LNPs (ES-PEI-LNPs). The surface charge of ES-PEI-LNPs switched from negative to positive under colonic conditions by the dissociation of the negatively charged ES layer. Kowsari et al. [137] fabricated nanocarrier that conjugated PEI and amino-bearing sericin to carboxylic-bearing fluorinated graphene oxide (FGO) by two different amide linkages. In this work, PEI could enhance cell internalization and nuclear-targeted delivery of curcumin. By anchoring sericin polypeptides onto the surface of PEI-FGO, this PEI-FGO-Sericin NPs exhibited step-by-step pH-dependent charge switch corresponding to extracellular tumor and endo/lysosome acidic environment via hydrolysis of the acid-labile amides, the subsequent electrostatic repulsion induced opening of the structure and aiding the endosomal release of the incorporated anticancer agents. Another interesting study about PEI-based charge reversal drug delivery is using PEI to prepare a microneedle patch for rapid gene release to treat subdermal tumor [173]. In this microneedle patch, PEI played as both gene-loaded support and charge-reversal layer. By coating with acid-sensitive polyelectrolyte multilayers (PEM), PEI-microneedle patch could achieve fast gene release when the system was exposed to an acidic environment in the tumor site after insertion.

## Summary and outlooks

In summary, CR-NDDSs with unique properties and advantages have been widely investigated for the treatment of different diseases, including tumor therapy. CR-NDDSs can be triggered to convert the surface charge by different stimuli, such as pH, enzymes, ROS, GSH and so on. Various types of CR-NDDSs—including nanoparticles, microneedles, micelles and so on—have been designed and fabricated to efficiently deliver the therapeutic agents. From the reported studies, CR-NDDSs show great potential for improving tumor targeting ability, enhanced cellular uptake and improved therapeutic efficiency. Table 3 summarizes some examples of the design and applications of CR-NDDSs for the enhanced therapy application.

**Table 3:** Summary of CR-NDDSs examples for tumor therapy

Mechanism for charge conversion	Materials	Therapeutic agent	Tumor model	Refs.
pH-responsive	PEG-DMMA	Ce6	HepG2	[50]
	PAH-DMMA	cisplatin	B16	[57]
	CMCS	DOX	HepG2	[60]
	PDEA	CPT	HeLa	[63]
	PBAE, HA	DOX	A549	[66]
	PHis	siRNA	A549	[67]
	PEOz-b-PSD, PAMAM	DOX	4T1	[72]
	p(MPC-ss-VIM)	DOX	A549	[78]
	CS-DMMA, PAMAM	DOX	HepG2	[132]
Enzyme-responsive	GluAcNA-Lip, β-glucuronic acid	LND	4T1	[19]
	PBEAGA, γ-glutamylamides	CPT	HepG2	[85]
	HA, TPP, PLLA	DOX, LND	4T1	[86]
ROS-responsive	BAP, DSPE-PCB	siRNA	GL261	[91]
	PEG, PCL, peroxalate esters	AQ4N	Hep3B	[93]
ATP-responsive	PBA, PEI, alginate	siRNA	4T1	[100]
GSH-responsive	HA, PAMAM	DOX	HeLa	[113]
H_2_S-responsive	PHEMA-b-PMMA	DOX	HeLa	[119]
pH/ROS-responsive	MPEG-PLL-DMA	PPT	A549	[125]
pH/GSH-responsive	PEG-PAH-DMA, PAMAM	PPT	A549	[135]

Although impressive therapeutic outcomes have been achieved using CR-NDDSs, there are still challenges for further application of CR-NDDSs. First, premature leakage of therapeutic agents and off-site accumulation are not yet solved. Unexpected leakage of therapeutic agents may increase the risk of toxic effects for healthy tissues, it also reduces therapeutic efficiency since lower amounts of therapeutic agents finally reach pathological regions. Second, the synthesis methods of CR-NDDSs should be more economic and eco-friendlier. During the synthesis process, most of reported CR-NDDSs are synthesized in organic solvents, expensive reagents are also used for obtaining the final drug delivery systems, which are unsuitable for large-scale production. Third, the rates of stimuli-triggered charge-conversion need to be optimized to meet the requirements of different therapy requirement. A proper charge-reversal rate could improve the cellular uptake and therapy efficiency, also lower the risk of unexpected side effects. Finally, more effort should be devoted to studying the fates of these CR-NDDSs in living bodies for the better understand of their potential clinical application prediction. Almost nanoparticles in the biological systems are wrapped by various biological proteins forming protein corona, which could alert the biological fate of nanoparticles, including the pharmacokinetics, biodistribution, and even therapeutic efficacy. Some protein corona may increase the targeting capability, while some leads to a decrease in the targeting efficiency since targeting ligand to the receptor of the cell membrane is shield. Protein corona also changes the drug release profile of nanocarriers. Therefore, more research work is needed to confirm the effects of protein corona and the biological consequences of CR-NDDSs.

In the future, research focus should shift to clinical application of using CR-NDDSs in disease treatment, not only cancer therapy, but also treatment of other diseases. There is still a long way to go before achieving clinical utility, we expect more therapeutics research and further understanding and development in this field in the near future.

## Abbreviations

ATP: Adenosine triphosphate
Azo: Azonzenecarboxylic acid
*β*-G: β-Glucuronidase
CD44: Cluster of differentiation 44
CMCS: Carboxymethyl chitosan
CPT: Camptothecin
CR-NDDSs: Charge reversal nano-drug delivery systems
CS: Chitosan
CSCs: Cancer stem cells
DCA: 1,2-Dicarboxylic-cyclohexene anhydride
DMMA: 2,3-Dimethylmaleic anhydride
DNA: Deoxyribonucleic acid
DOX: Doxorubicin
EPR: Enhanced permeability and retention
GGT: γ-Glutamyl transpeptidase
GSH: Glutathione
H_2_S: Hydrogen sulfide
HA: Hyaluronic acid
HAase: Hyaluronidase
HAS: Human serum albumin
MDR: Multidrug resistance
NDDSs: Nano-drug delivery systems
NIR: Near-infrared light
PAMAM: Poly(amidoamine)
PBAEs: Poly(beta-amino esters)s
PCL: Poly(caprolactone)
PDEA: Poly(2-(diethylamino)ethyl methacrylate)
PEG: Poly(ethylene glycol)
pHe: Extracellular pH
Phis: Poly(histidine)
PLL: Polylysine
PO: Peroxalate esters
PSD: Poly(methacryloyl sulfadimethoxine)
ROS: Reactive oxygen species
TM: Tetramethyl succinic anhydride
TME: Tumor microenvironment
TNBC: Triple negative breast cancer
VEGF: Vascular endothelial growth factor
